# Antiangiogenic Potential of Pomegranate Extracts

**DOI:** 10.3390/plants13233350

**Published:** 2024-11-29

**Authors:** Riccardo Tornese, Anna Montefusco, Rocco Placì, Teodoro Semeraro, Miriana Durante, Monica De Caroli, Gianpiero Calabrese, Anna Eleonora Caprifico, Marcello Salvatore Lenucci

**Affiliations:** 1 Dipartimento di Scienze e Tecnologie Biologiche ed Ambientali (DiSTeBA), Università del Salento, Via Prov.le Lecce Monteroni, 73100 Lecce, Italy; riccardo.tornese1@unisalento.it (R.T.); anna.montefusco@unisalento.it (A.M.); rocco.placi@unisalento.it (R.P.); monica.decaroli@unisalento.it (M.D.C.); 2 Research Institute on Terrestrial Ecosystems (IRET-URT Lecce), National Research Council of Italy (CNR), Campus Ecotekne, 73100 Lecce, Italy; teodoro.semeraro@cnr.it; 3 Istituto di Scienze delle Produzioni Alimentari (ISPA)-CNR, Via Prov.le Lecce-Monteroni, 73100 Lecce, Italy; miriana.durante@ispa.cnr.it; 4 NBCF National Biodiversity Future Center, 90133 Palermo, Italy; 5 School of Life Sciences, Pharmacy and Chemistry, Kingston University London, Penrhyn Road, Kingston upon Thames, London KT1 2EE, UK; 6 School of Allied Health Sciences, Faculty of Health and Life Sciences, De Montfort University, The Gateway, Leicester LE1 9BH, UK

**Keywords:** *Punica granatum* L., angiogenesis, punicalagin, ellagic acid, ellagitannins, extraction methods, punicic acid, cancer, supplement, VEGF

## Abstract

Pomegranate (*Punica granatum* L.) has long been recognised for its rich antioxidant profile and potential health benefits. Recent research has expanded its therapeutic potential to include antiangiogenic properties, which are crucial for inhibiting the growth of tumours and other pathological conditions involving aberrant blood vessel formation. This review consolidates current findings on the antiangiogenic effects of pomegranate extracts. We explore the impact of pomegranate polyphenols, including ellagic acid, punicalagin, anthocyanins, punicic acid and bioactive polysaccharides on key angiogenesis-related pathways and endothelial cell function. Emphasis is placed on the effects of these extracts as phytocomplexes rather than isolated compounds. Additionally, we discuss the use of pomegranate by-products, such as peels and seeds, in the preparation of extracts within a green chemistry and circular economy framework, highlighting their value in enhancing extract efficacy and sustainability. By primarily reviewing in vitro and in vivo preclinical studies, we assess how these extracts modulate angiogenesis across various disease models and explore their potential as adjunctive therapies for cancer and other angiogenesis-driven disorders. This review also identifies existing knowledge gaps and proposes future research directions to fully elucidate the clinical utility of pomegranate extracts in therapeutic applications.

## 1. Introduction

### 1.1. Understanding Angiogenesis and Its Key Regulatory Processes

Angiogenesis is the process by which new blood vessels form from pre-existing ones. It plays a crucial role in embryonic development, tissue repair, and physiological processes like wound healing. However, when dysregulated, abnormal angiogenesis—whether excessive or insufficient—can contribute to a variety of diseases, including cancer, atherosclerosis, arthritis, psoriasis, endometriosis, obesity and SARS-CoV-2 [1].

The following three key processes shape the vascular system:

– Vasculogenesis: During embryogenesis, endothelial precursor cells (angioblasts) differentiate into endothelial cells, leading to the de novo development of blood vessels.
– Angiogenesis: In adults and embryos, new blood vessels sprout from existing small vessels (sprouting angiogenesis) or form through intravascular subdivision (intussusception). Pruning and remodelling transform the existing vasculature into a mature network.
– Arteriogenesis: The rapid proliferation of pre-existing collateral vessels (arterioles) transforms them into functional arteries. The process plays an important role in enhancing perfusion to collateral-dependent ischemic regions.

Once these three processes are completed during postnatal development, the adult vasculature becomes stable and rarely proliferates under physiological conditions.

**Figure 1:**
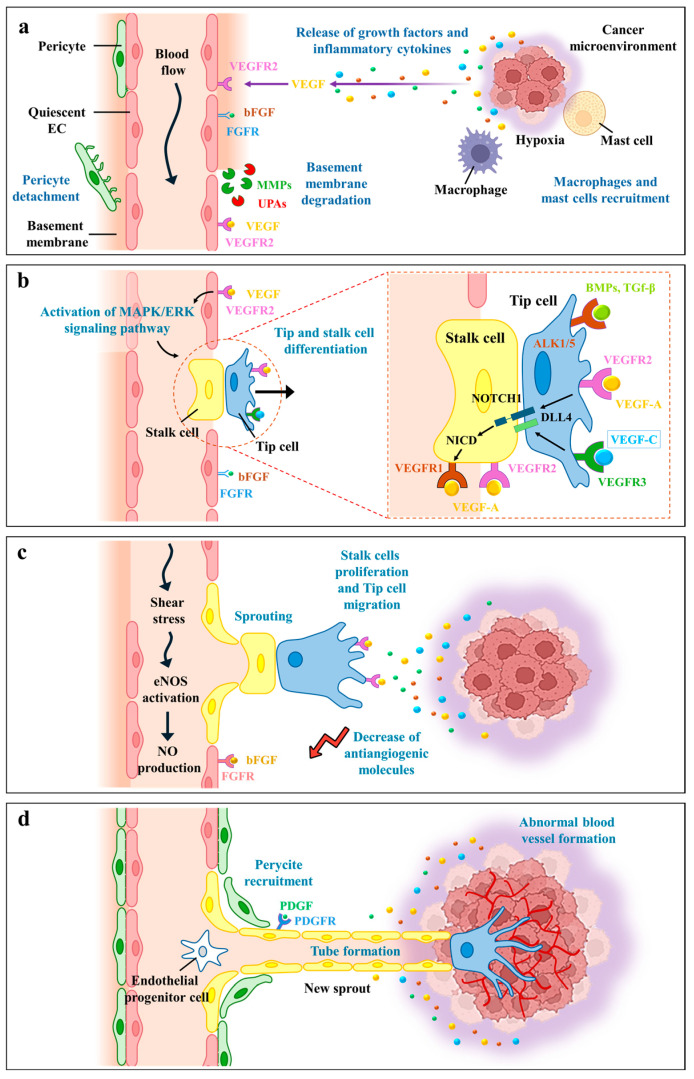
Schematic illustration of key steps in angiogenesis and multicellular interactions during new vessel development. (**a**) In rapidly growing tumours, severe hypoxia leads to the stabilisation of HIF proteins (HIF-1α and HIF-2α), triggering the synthesis and release of proangiogenic factors like VEGFs. These factors are initially sequestered by the basement membrane (BM) and cannot reach their target receptors on endothelial cell (EC) membranes. Cancer cells recruit macrophages and mast cells from the nearby stroma, which release matrix metalloproteinases (MMPs) that start degrading BM. (**b**) Degradation of BM releases sequestered proangiogenic factors, allowing them to bind to their receptors and activate the MAPK/ERK signalling pathway. Phosphorylated ERK translocates to the nucleus to regulate transcription, promoting EC proliferation and migration. Additionally, increased endothelial permeability releases more MMPs and urokinase plasminogen activators (UPAs), participating in activating pericellular proteolysis and releasing additional proangiogenic factors (e.g., bFGF, TGF-β, PDGF, angiogenin and angioprotein). Activin receptor-like kinases (ALK1/5) work together to regulate angiogenesis, with ALK1 promoting the formation of new vessels and ALK5 contributing to their maturation and stabilisation. VEGF-DLL4/Notch signalling regulates the formation of tip and stalk cells: VEGF induces DLL4 expression in tip cells, activating Notch1 signalling and suppressing tip cell formation in adjacent stalk cells. Tip cells adhere to the extracellular matrix via integrins, start to form lamellipodia and filopodia and migrate toward guidance signals like semaphorins and ephrins. (**c**) Stalk cells proliferate behind the tip cell, elongating the sprout along the VEGF gradient towards the tumour microenvironment and initiating lumen formation. Growth factors also decrease the levels of antiangiogenic molecules (e.g., thrombospondins, angiostatin, endostatin), while eNOS and NO synthesis in endothelial cells under shear stress enhance blood flow, vascular permeability, and endothelial cell proliferation and migration. NO also interacts with VEGF, amplifying its angiogenic effects. (**d**) Proangiogenic signals recruit endothelial progenitor cells from the bone marrow, accelerating vascularisation. As sprouting continues, stalk cells deposit BM and recruit pericytes, stabilising the forming vessel. Pericyte precursors are attracted by endothelial cell-expressed PDGF and differentiate into mature pericytes in response to TGF-β, reducing endothelial cell migration, proliferation, and vascular leakage, leading to vessel stabilisation and the formation of mature vasculature. HIFs, Hypoxia-Inducible Factors; VEGFs, Vascular Endothelial Growth Factor; ERK, Extracellular signal-Regulated Kinase; bFGF, basic Fibroblast Growth Factor; TGF-β, Transforming Growth Factor beta; PDGF, Platelet-Derived Growth Factor; DLL4, Delta-Like ligand 4; eNOS, endothelial Nitric Oxide Synthase; NO, Nitric Oxide.

Angiogenesis is tightly regulated by a balance between pro-angiogenic and antiangiogenic factors (Figure 1). Among the pro-angiogenic factors, Hypoxia-Inducible Factors (HIFs), Platelet-Derived Growth Factors (PDGFs), Vascular Endothelial Growth Factors (VEGFs) and VEGF-like molecules play a pivotal function. When VEGF binds to its receptor, VEGFR-2, it initiates a signalling cascade that leads to endothelial cell activation, sprouting, and increased vessel permeability [2,3].

This binding also triggers the PI3K/AKT/mTOR pathway, leading to the upregulation of the pro-survival B-cell lymphoma 2 (Bcl-2) protein, which protects endothelial cells from apoptosis. The balance between pro-apoptotic Bcl-2-associated X protein (Bax) and anti-apoptotic Bcl-2, indicated by the Bax/Bcl-2 ratio, is central to angiogenesis, affecting endothelial cell proliferation and vessel stability. Additionally, Bcl-2 activates Nuclear Factor-*κ*B (NF-*κ*B), which upregulates pro-angiogenic chemokines CXC Ligand-1 (CXCL1) and CXC Ligand-8 (CXCL8). This enhances the angiogenic potential of endothelial cells through autocrine signalling via the CXC Receptor-2 (CXCR2) [4]. NF-*κ*B also induces the expression of the AP-1 subunit JunB and acts as a transcriptional partner of JunB to modulate the expression of genes associated with the inflammatory response. A reduction of JunB in various cell types results in severely impaired hypoxia-induced VEGF expression, despite the presence and stabilisation of HIFs [5].

Another key factor, the basic Fibroblast Growth Factor (bFGF), is instrumental in promoting the proliferation and survival of endothelial cells. Additionally, Intercellular Adhesion Molecules (ICAMs) and Vascular Cell Adhesion Molecules (VCAMs), along with Matrix Metalloproteinases (MMPs), facilitate the migration of cells required for the formation of new blood vessels [6,7]. On the other hand, antiangiogenic factors (i.e., thrombospondins, angiostatin and endostatin) inhibit angiogenesis and help maintain the quiescence of the vascular system [8]. To trigger angiogenesis, the production of activators must increase while the production of inhibitors decreases. Understanding these molecular mechanisms is essential for developing therapeutic approaches targeting angiogenesis in various diseases. Furthermore, reduced angiogenesis may result from the proteolytic breakdown of pro-angiogenic factors, a process commonly observed during wound healing in certain pathological conditions, such as diabetic ulcers [9].

### 1.2. Angiogenesis and Cancer: A Dynamic Interplay

Cancer growth is intricately linked to angiogenesis. This phenomenon is not just a hallmark of cancer but a fundamental enabler of tumour survival and expansion. Tumours, much like normal tissues, require a steady supply of oxygen and nutrients to sustain their rapid proliferation. Angiogenesis fulfils this need by developing an extensive vascular network that infiltrates the tumour, providing the necessary sustenance and even a route for metastasis, where cancer cells spread to other parts of the body [10,11]. Over a dozen proteins and a variety of smaller molecules have been identified as “angiogenic” signals, released by tumours to kickstart angiogenesis. Key players in this process include VEGF and bFGF. These angiogenic factors act as growth signals, prompting endothelial cells to not only divide and move towards the source of the signal but also to transform into complex tubular structures essential for new blood vessel formation [12]. Tumour-induced inflammation and oxidative stress also participate in initiating pathological angiogenesis. When macrophages and neutrophils infiltrate the tumour microenvironment, they release pro-inflammatory cytokines, chemokines and Reactive Oxygen Species (ROS). These factors stimulate the production of angiogenic molecules, including VEGF and bFGF, leading to the development of abnormal vasculature. Furthermore, chronic inflammation and oxidative stress contribute to the creation of a hypoxic tumour environment, which activates signalling pathways like MAPK/ERK and stabilises hypoxia-inducible factor-1 alpha (HIF-1α), further upregulating the expression of angiogenic factors. This interplay between inflammation, oxidative stress and angiogenesis not only promotes tumour growth but also contributes to metastasis, becoming a significant area of study for developing anti-cancer therapies [13].

The concept of targeting angiogenesis for cancer therapy emerged in the 1970s, pioneered by Judah Folkman, who hypothesised that cutting off a tumour’s blood supply could inhibit its growth. This revolutionary idea shifted the paradigm of cancer treatment, leading to the development of antiangiogenic drugs designed to thwart the formation of new blood vessels [14]. These therapies aim to starve the tumour by blocking the signalling pathways that promote vascular growth, such as the VEGF pathway. An example of an antiangiogenic agent used in clinical practice since 2004 is Bevacizumab (Avastin, developed by Genentech), a humanised recombinant monoclonal antibody directed toward VEGF (rhu Anti-VEGF Mab) [15]. Despite the theoretical promise of antiangiogenic strategies, their practical application has faced challenges [16]. One significant issue is that these therapies are often administered after cancer has already progressed, by which time the tumour has developed sophisticated mechanisms to adapt and survive. Additionally, tumours can become resistant to antiangiogenic treatments, finding alternative pathways to continue their growth and spread [17]. Moreover, the dynamic interplay between angiogenesis and cancer is not a one-way street. Tumours actively influence their surroundings, creating an environment that fosters angiogenesis. They release various growth factors and enzymes that degrade the extracellular matrix, making it easier for new blood vessels to sprout (Figure 1). This bidirectional interaction highlights the complexity of the tumour microenvironment and underscores the importance of understanding the various factors that promote or inhibit angiogenesis. It also emphasises the need for more nuanced approaches to overcoming cancer resistance mechanisms, particularly in relation to antiangiogenic therapies [18].

Recent research has also explored the potential of integrating antiangiogenic therapies with other treatment modalities. For instance, combining these therapies with immunotherapy, which harnesses the body’s immune system to fight cancer, has shown promising results. By disrupting the blood supply to the tumour, antiangiogenic drugs can enhance the efficacy of immunotherapies, making it harder for the tumour to evade immune detection and destruction [19].

### 1.3. Shifting Paradigms in Cancer Prevention: The Role of Natural Antiangiogenic Agents

Several years ago, researchers observed that certain cancer chemo-preventive agents exhibited antiangiogenic properties, shifting cancer research from treatment to prevention. This discovery highlighted the strategy of targeting angiogenesis to inhibit tumour growth from the outset. The concept of prevention encompasses a wide array of possibilities, from dietary modifications and lifestyle changes to the use of repurposed drugs known to inhibit angiogenesis [11]. Compounds in everyday foods like epigallocatechin, a flavonoid abundant in green tea, and resveratrol in red grapes have shown promise in preventing angiogenesis. These natural substances not only boost overall health but also disrupt the formation of new blood vessels, thereby starving potential tumours of their vital nutrients and oxygen. Omega-3 fatty acids, known for cardiovascular benefits, are now recognised for their role in cancer prevention by interfering with tumour vascular supply. Additionally, retinoids—especially fenretinide—and carotenoids like lycopene and fucoxanthin are under investigation for their antioxidant properties, cardioprotective effects, and potential anticancer benefits [20,21]. Even common medications such as aspirin, β-blockers, and renin-angiotensin-aldosterone system inhibitors are being re-evaluated for their potential to inhibit blood vessel formation and offer new avenues for cancer prevention.

This shift towards prevention highlights the potential of combining dietary elements, lifestyle adjustments, and established medications in a comprehensive strategy to fight cancer. By focusing on early detection and intervention, and expanding our understanding of angiogenesis inhibition, we can drastically reduce the incidence and severity of cancer and other angiogenesis-dependent diseases. This approach provides a proactive and holistic method for health management. With ongoing research and increased awareness, harnessing natural compounds holds the potential to revolutionise cancer prevention, elevating its importance to that of treatments [22].

## 2. Pomegranate: A Multifunctional Natural Powerhouse in Cancer Prevention

**Figure 2:**
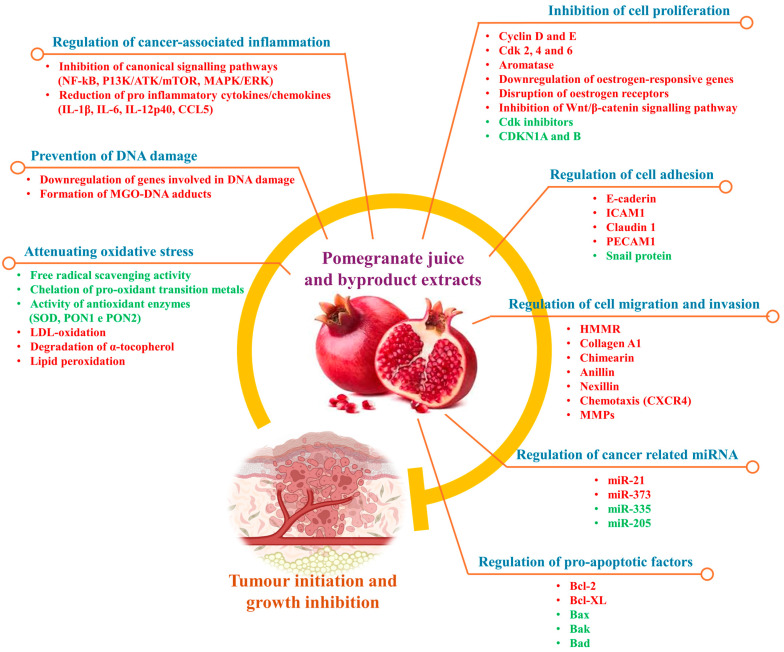
Potential inhibitory mechanisms of pomegranate juice and extracts on cancer initiation and progression. Processes that are upregulated or activated are shown in green, while those that are downregulated or inhibited are shown in red. Bad, Bcl-2-associated death promoter; Bak, Bcl-2 homologous antagonist/killer; Bax, Bcl-2-associated X protein; Bcl-2; B-cell lymphoma 2 protein; Bcl-XL, B-cell lymphoma-Extra Large protein; CCL5, C-C motif chemokine ligand 5; CDK, Cyclin-dependent kinase; CXCR4, C-X-C chemokine receptor type 4; E-caderin, Epithelial caderin; HIF-1α, Hypoxia-Inducible Factor 1-alpha; HMMR, Hyaluronan-Mediated Motility Receptor; ICAM1, Intercellular Adhesion Molecule 1; ILs, interleukins; LDL, Low-Density Lipoprotein; KIP1/P27, Cyclin-dependent kinase inhibitor 1B; MGO, methylglyoxal; miRs, microRNAs; MMPs, Matrix metalloproteinases, PON, Paraoxonase; PECAM1, Platelet Endothelial Cell Adhesion Molecule; SOD, Superoxide Dismutase; VEGF, Vascular Endothelial Growth Factor; CDKNs, Cyclin-dependent kinase inhibitors.

Plants have a long-standing history in cancer therapy, with nearly three-quarters of anti-tumour compounds used in modern medicine being natural products or their derivatives [23]. Among cancer-preventing plants, pomegranate (*Punica granatum* L.) stands out for its ability to interfere with tumour initiation and progression through various mechanisms, including the modulation of inflammation, induction of apoptosis, inhibition of cell proliferation and invasion (metastasis), and, relevant to this review, prevention of angiogenesis (Figure 2) [24].

Rich in polyphenols like ellagic acid and punicalagin, pomegranates effectively inhibit the formation of new blood vessels necessary for tumour growth. This antiangiogenic effect, combined with its strong antioxidant properties, makes pomegranate and its by-product extracts valuable additions to a cancer-preventive diet [25,26]. Key molecular targets influenced by pomegranate include Bax/Bcl-2 ratio, cyclins, VEGF, MMPs and Peroxisome Proliferator-Activated Receptors (PPARs) [27,28]. However, the antiangiogenic effect and related molecular targets of pomegranate extracts have been demonstrated primarily in vitro and in animal cancer models. While in vivo studies have shown no significant cytotoxicity following oral administration of pomegranate extracts, no clinical trials have yet evaluated their efficacy as antiangiogenic agents. This gap may be attributed to the absence of systemic toxicity data related to the intravenous administration of pomegranate extracts, highlighting the need for further investigation.

Finally, it remains poorly investigated the effect of high ingestion of pomegranate in patients experiencing high angiogenesis in conditions such as pregnancy where these antiangiogenic effects might be deleterious for the development of the fetus.

Notably, pomegranate consumption has generally been shown to be safe; for instance, a study involving 86 overweight human individuals reported no adverse effects after administering 1420 mg/day of pomegranate fruit extract in tablet form for 28 days [29,30,31]. However, the effects of high pomegranate intake in conditions characterised by elevated angiogenesis, such as pregnancy, remain poorly understood. In such cases, the antiangiogenic properties of pomegranate could potentially have adverse effects on fetal development, underscoring the need for targeted research in this area.

### 2.1. Anatomy, Composition and Nutritional Benefits of Pomegranate Fruit

**Figure 3:**
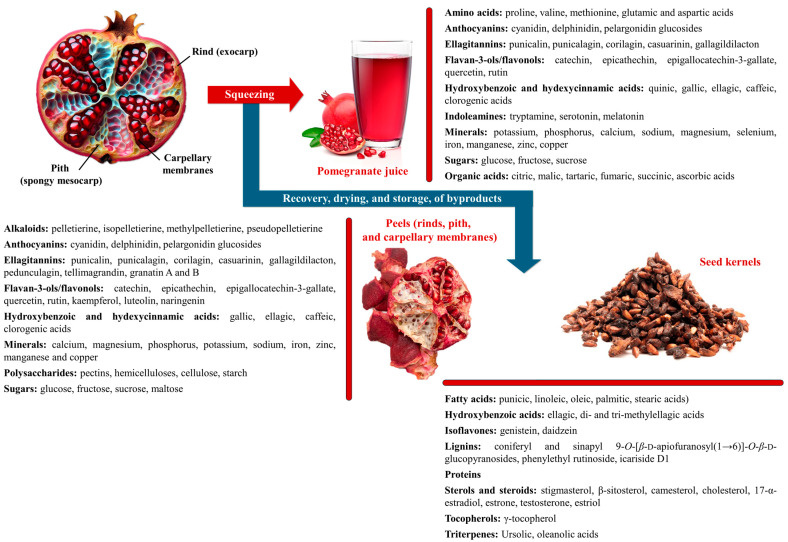
Pomegranate fruit anatomy and key chemical constituents identified in the juice, peel and kernel fractions.

The pomegranate fruit consists of three main parts: the pericarp, also known as the husk or rind, which is the outer-coloured leathery shell; the mesocarp, or albedo, which comprises the inner spongy pith and the yellowish, papery, septal (carpellary) membranes; and the edible arils (seeds), which contains the sarcotesta from which the juice is obtained, along with the kernel, the woody part of the seed. Each portion contains beneficial compounds (Figure 3) [32].

The juice is rich in water, macro and microelements, carbohydrates, pectins, organic acids, several polyphenols, and other compounds such as fatty acids, amino acids and tocopherols. Its intense red pigmentation results mainly from the presence of anthocyanins and ellagitannins [33]. The peels, comprising rind, pith and septal membranes, contain minerals, carbohydrates, polyphenols, dietary fibres, fatty acids, organic acids and alkaloids [34,35]. Among these, polyphenols, particularly hydrolysable tannins, such as ellagitannins and gallotannins, found predominantly in the pith and membranes, significantly contribute to pomegranate’s antioxidant capacity and are the most relevant for pharmaceutical applications [36]. Pomegranate kernel oil, rich in punicic acid (73.4–83.5% of total fatty acids), also contains tocopherols, phytosterols, cerebrosides, and both steroidal and non-steroidal oestrogenic phytochemicals [37].

### 2.2. Anti-Inflammatory, Antioxidant and Neuroprotective Effects of Pomegranate Extracts

Pomegranate extracts, especially those high in punicic acid and ellagitannins, exhibit potent anti-inflammatory effects by inhibiting NF-κB, PI3K/AKT/mTOR and MAPK/ERK signalling pathways and downregulating cyclooxygenases (COX-1 and COX-2), and microsomal Prostaglandin E Synthase-1 (mPGES-1) expression. This results in reduced production of prostaglandins and a decrease in the activity of inflammatory cytokines, which are significant in various pathologies, including those associated with dysregulated angiogenesis [38,39]. Besides, the high polyphenol content in pomegranates provides substantial antioxidant benefits, reducing oxidative stress, DNA damage and lipid peroxidation. Compounds such as punicalagin and anthocyanidins efficiently scavenge reactive oxygen and nitrogen species, enhancing overall cellular health [40,41]. Pomegranate extracts have also demonstrated neuroprotective effects by lowering soluble human amyloid beta (Aβ42) peptide levels and reducing amyloid deposition in the hippocampus, which is relevant for improving Alzheimer’s disease outcomes and enhancing memory following ischemia [42]. Additionally, studies suggest that Aβ42 can disrupt normal angiogenesis by either promoting abnormal vessel growth or inhibiting new vessel formation. This dysregulation may contribute to the progression of Alzheimer’s disease and other conditions where angiogenesis plays a critical role, such as cancer and age-related macular degeneration [43,44].

## 3. Sustainable Extraction and Valorisation of Pomegranate Bioactive Compounds in a Circular Economy

**Figure 4:**
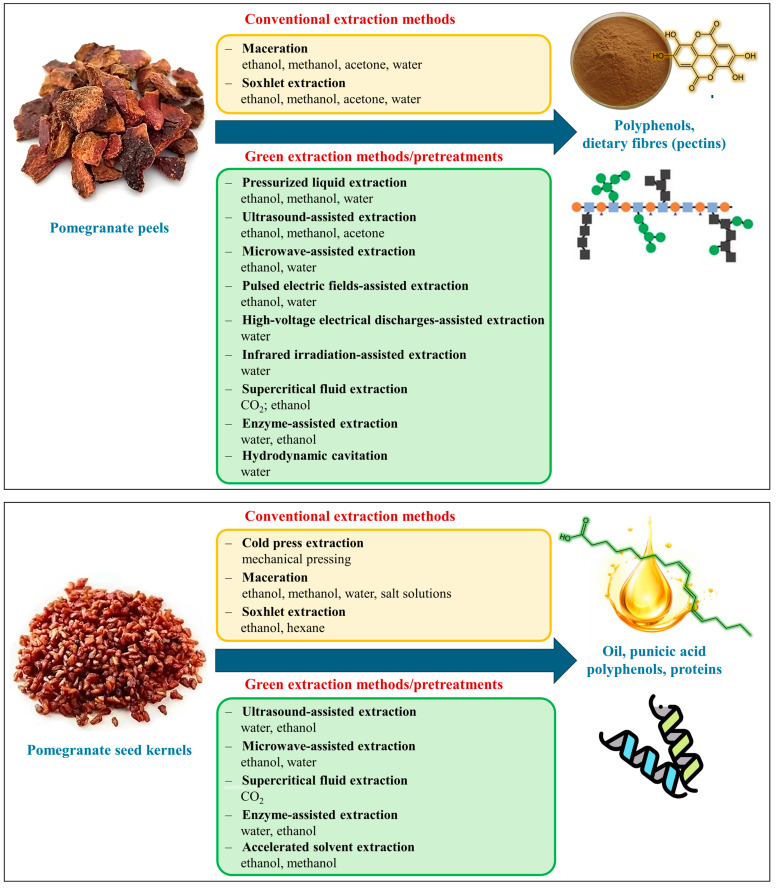
Valorisation of bioactive compounds from pomegranate peels and seed kernels using conventional and green extraction methods.

Pomegranate bioactive compounds, particularly those found in fruits and their by-products like peels and kernels, are extracted using both traditional and green techniques (Figure 4).

Traditional juice extraction methods typically involve mechanical pressing, ranging from aril extraction without kernel crushing to pressing whole or halved fruits using squeezers or rack-and-cloth presses. Recent advancements have integrated non-edible pomegranate parts—such as peels, carpellary membranes, and pith—into juice extraction processes. This approach enhances the juice with higher concentrations of bioactive compounds, though it may alter the flavour [45].

Globally, pomegranate production reaches approximately 3 million tons annually, with rinds and kernels comprising about 54% of the fruit. This translates to an estimated 1.62 million tons of waste, presenting a significant opportunity for valorisation within a circular economy framework that emphasises waste reduction and resource efficiency [46].

Traditional methods for recovering bioactive molecules from pomegranate by-products, such as maceration, Soxhlet extraction and cold pressing, are still widely used. These techniques are particularly common for extracting polyphenols from peels using methanol or ethanol and for extracting oil from kernels. However, some traditional methods raise sustainability concerns due to the use of potentially toxic solvents and the risk of degrading thermolabile nutritional compounds during processing [47]. For instance, while cold pressing of kernel oil preserves its nutritional quality without the use of solvents, the yields are often lower compared to solvent extraction [48]. To address these challenges, green extraction methods have emerged as more sustainable alternatives. Techniques such as ultrasound-assisted extraction, microwave-assisted extraction, enzyme-assisted extraction, hydrodynamic cavitation, and sub- or supercritical fluid extraction have gained popularity. These methods reduce energy consumption and solvent use while preserving the integrity of bioactive compounds. Despite their environmental benefits and effectiveness in maintaining compound stability, they still face challenges in scalability and standardisation for industrial applications [45]. Additionally, the proper storage and preservation of pomegranate peels, seeds, juices and extracts are essential to prevent oxidative deterioration and microbial growth. Effective pre-treatment processes, including drying, homogenization, and grinding into powder, are crucial for optimising extraction quality. These processes must be carefully selected and fine-tuned to minimise the degradation of bioactive compounds and ensure stability during storage [46].

Emerging green pre-treatment technologies, such as cell permeabilization methods involving pulsed electric fields, high-intensity ultrasound, and microwave heating, can greatly enhance extraction efficiency. Integrating these technologies into the biorefinery of pomegranate by-products improves mass transfer and facilitates solvent access to target compounds. These strategies can be applied individually or in combination to optimise sequential extraction processes, leading to the development of valuable new ingredients for use in food, cosmetics and pharmaceuticals [49].

In recent years, sequential biorefinery extraction methods have attracted considerable attention to improve the efficiency and sustainability of biomass processing. These methods systematically recover valuable compounds through multiple extraction steps, each designed to target specific molecules in the raw material [50]. For instance, a two-step process combining expeller pressing with supercritical CO₂ extraction has proven effective for extracting pomegranate kernel [51]. Additionally, a one-pot enzymatic green process utilising protease from *Aspergillus oryzae*, followed by centrifugation to separate the aqueous phase, solid residue and oil, has been explored to recover high-quality oil, food-grade proteins and fibres from pomegranate kernel waste. This process yielded oil with higher levels of conjugated fatty acids, polyphenols and antioxidant activity, while the extracted proteins were rich in all essential amino acids [52].

A comprehensive biorefinery approach has also been employed to extract key products such as ellagic acid, lignin and pectin from pomegranate peels [53]. Moreover, natural deep eutectic solvents and pressurised liquids have been used sequentially to extract proteins from defatted pomegranate kernels [54]. The primary advantage of sequential extraction methods is their ability to enhance the economic viability of the process while simultaneously reducing greenhouse gas emissions associated with each product. This results in increased process efficiency and a lower environmental impact.

Once obtained, pomegranate extracts can be incorporated into various food matrices to enhance “clean label” products and improve functional properties, aligning with current trends in health, well-being and sustainability [55]. Leveraging these by-products allows the pomegranate industry to optimise production costs and develop affordable, value-added products, such as dietary supplements. This approach not only contributes to a more sustainable and circular economy but also supports the discovery of novel phytocomplexes with potential therapeutic applications.

## 4. Bioactive Compounds in Pomegranate: Antiangiogenic and Therapeutic Potential

Pomegranate fruit is abundant in bioactive compounds that exhibit promising antiangiogenic properties. Notable molecules include ellagitannins (such as punicalagin, punicalin and ellagic acid), flavonoids including anthocyanins, flavonols (quercetin and kaempferol) and flavones (luteolin glycosides), fatty acids and polysaccharides. These compounds also exhibit antioxidant and anti-inflammatory activities, further contributing to their antiangiogenic effects [38].

### 4.1. Ellagitannins

**Figure 5:**
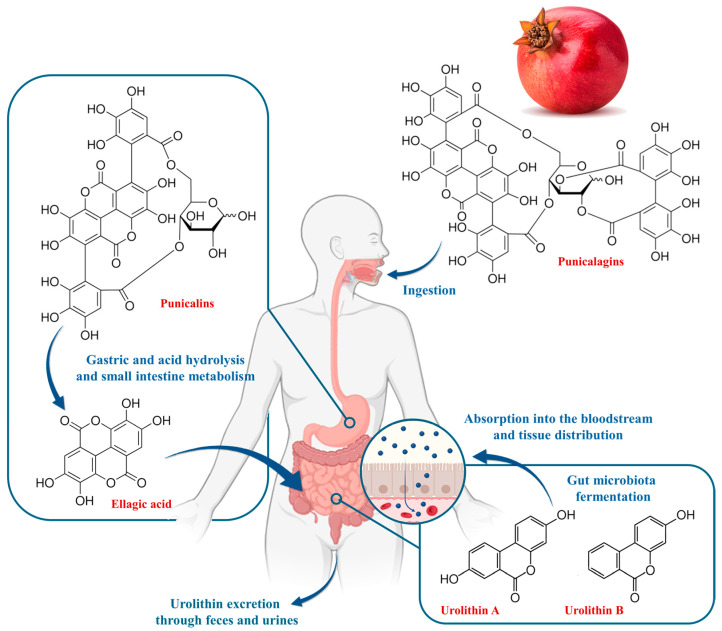
Metabolic pathway of punicalagin to urolithins in the human digestive system. Ingested punicalagin is initially hydrolysed in the stomach and small intestine to produce punicalin and ellagic acid. These compounds are then transformed by the gut microbiota through a series of reactions, leading to the formation of various urolithins, with urolithin-A and urolithin-B being two key final products.

Ellagitannins, a subgroup of hydrolysable tannins formed by the combination of gallic acid and hexahydroxy diphenic acid with glucose, exhibit diverse structures, including glycosides of ellagic acid. Numerous data suggest that ellagitannins exhibit a wide range of biological and clinically relevant activities and have the potential for health promotion and medical applications, including cancer prevention and treatment [56]. However, due to the complexity of their structure, most of the ellagitannins from various sources are not absorbed in the human gastrointestinal system. Therefore, the strong bioactivity of dietary ellagitannins can be explained by their ability to be hydrolysed in the digestive system, primarily to ellagic acid and other smaller polyphenols, and also to produce biologically active metabolites in vivo. At the same time, the health-promoting effects of ellagitannins are also related to their inherent biological activity in addition to the effects of their breakdown products (Figure 5).

Prominent among ellagitannins is punicalagin, found abundantly in pomegranate juice, peels, seeds, flowers, leaves, bark and roots. It exists in two anomeric forms, α and β, with concentrations in pomegranate juice reported at 95 mg/g and 43 mg/g, respectively, while ellagic acid is present at around 13 mg/g [57]. Upon ingestion, punicalagin hydrolyses to release ellagic acid, gallagic acid dilactone and D-glucose [38]. Punicalagin exhibits a broad spectrum of bioactive properties, including anti-inflammatory, antioxidant and anti-proliferative/pro-apoptotic effects. It reduces inflammation in various cell types, including macrophages (RAW264.7), microglia (BV2), macroglia and liver cells (HepG2), by modulating inflammation-associated gene expressions and inhibiting the MAPK/ERK and NF-κB signalling pathways.

As an antioxidant, punicalagin protects PC12 neuronal cells from oxidative stress induced by hydrogen peroxide, mitigating damage from ROS. Punicalagin’s anti-proliferative effects span multiple cancer cell lines, including lung cancer (A549), osteosarcoma (U2OS, MG63, SaOS2), breast cancer (MCF7, MDA-MB-231), colorectal cancer (HT-29, HCT-116, Caco-2), leukaemia (THP-1), thyroid carcinoma (BCPAP), cervical cancer (HeLa, ME-180) and pancreatic cancer (PANC-1, Aspc-1). These effects are mediated through apoptosis regulation, with increased expression of pro-apoptotic markers like Bax and decreased expression of anti-apoptotic markers such as Bcl-XL and Bcl-2. Additionally, punicalagin enhances the mRNA levels of Nrf2 (Nuclear factor erythroid 2-related factor 2), which regulates cellular antioxidative responses by influencing target genes like HO-1, GCLC, GCLM, GSTM1, GSTA4 and NQO-1, bolstering the cell’s antioxidative defences. Punicalagin demonstrates potent antiangiogenic activity, primarily through its effects on endothelial and cancer cells. Studies have shown that punicalagin can inhibit the proliferation and migration of human umbilical vein endothelial cells (HUVECs), which are critical for angiogenesis. This inhibition is associated with a decrease in the expression of VEGF and other pro-angiogenic factors [58]. In osteosarcoma cell lines U2OS and SaOS2, punicalagin reduces the production of interleukins IL-6 and IL-8, which are involved in promoting angiogenesis via the NF-κB signalling pathway [59]. Moreover, in xenografted mice models, punicalagin injection attenuated the growth of osteosarcoma and reduced tumour neoangiogenesis [60].

Under certain conditions, punicalagin can be hydrolysed to punicalin and ellagic acid. Punicalin also exhibits several health benefits including anti-inflammatory, antioxidant and antiviral activities. Its antioxidant properties help reduce the risk of various diseases, such as nephrotoxicity, cancer, diabetes, inflammation, liver disease and neurological conditions. Notably, punicalin has demonstrated neuroprotective effects, showing promise in mitigating neuroinflammation, improving learning and enhancing memory function in animal models. Specifically, punicalin has been found to inhibit LPS-induced memory impairment through anti-inflammatory and anti-amyloidogenic mechanisms by inhibiting Toll-Like Receptor-4 (TLR4)-activated NF-κB signalling [61]. Additionally, punicalin (10 mg/kg, intraperitoneal injection) alleviates LPS-induced acute lung injury in mice by reducing the production of inflammatory cytokines and inhibiting MAPK/ERK and NF-κB signalling [62]. While more research is needed to fully understand its bioavailability and mechanisms of action, punicalin holds potential as a therapeutic agent for neurological health and angiogenesis regulation.

Ellagic acid, the predominant polyphenol in pomegranates, typically exists conjugated with glucose or as part of ellagitannins [56]. Upon hydrolysis, it exhibits antiangiogenic, anti-inflammatory, antioxidant and anti-tumour properties [38]. Known for its potent antioxidant properties, it inhibits the formation of advanced glycation end products and free radicals, contributing to its wide range of bioactive effects as well as antiangiogenic properties. Ellagic acid inhibits steps of angiogenesis by targeting VEGFR-2, inhibiting its tyrosine kinase activity and downstream signalling pathways (such as MAPK/ERK and PI3K/AKT/mTOR), thereby impeding endothelial cell proliferation, migration and tube formation. Ellagic acid demonstrated an antiangiogenic effect against breast cancer cells MDA-MB-231, hepatocellular carcinoma, bladder cancer cells T24, UM-UC-3, 5637, HT-1376 and HUVECs. It also reduces neo-vessel formation in the chick embryo chorioallantoic membrane (CAM) and inhibits sprout formation in the chicken aorta [63,64,65,66]. Conversely, urolithins, particularly urolithin A, which result from the gut microbiota’s catabolism of ellagic acid, play a significant role in promoting angiogenesis. Urolithin A has been shown to enhance angiogenic pathways in skeletal muscle by increasing ATP and NAD^+^ levels—both essential for cellular energy and metabolism—while inhibiting the PI3K/AKT/mTOR pathway. Furthermore, it activates the SIRT1-PGC-1α pathway, a crucial cellular signalling mechanism for regulating energy metabolism and mitochondrial biogenesis. This activation leads to the upregulation of angiogenic markers such as VEGFA and CDH5, a member of the VCAM family [67,68].

Ellagic acid also demonstrates anti-tumour effects against various cancer cell lines, including osteosarcoma (Saos-2, MG63), breast cancer (MCF-7), ovarian cancer (ES-2, PA-1, A2780), melanoma (WM115), lung cancer (A549), prostate adenocarcinoma, glioblastoma (U251 MG, U87, MG, U118), oral cancer (HSC-2) and colorectal carcinoma (Caco-2). Its anti-inflammatory properties involve downregulation of iNOS, COX-2, TNF-α and IL-6 through NF-κB inhibition, and chemopreventive effects via the PI3K/AKT/mTOR pathway [38]. Combined with punicalagin and punicic acid, ellagic acid enhances anti-inflammatory and antioxidant effects, as evidenced in cardiac fibroblasts (IM-HCF) under TGF-β1-induced inflammation [69]. Several lines of evidence indicate that oestrogen directly modulates angiogenesis by acting on endothelial cells. Ellagic acid may exhibit both oestrogenic and anti-oestrogenic activities, depending on which oestrogen receptor it binds to. Specifically, binding to the oestrogen-alpha receptor (ERα) triggers oestrogenic effects, while binding to the oestrogen-beta receptor (ERβ) leads to anti-oestrogenic activity. As a result, ellagic acid can differentially influence angiogenesis, with its effects varying according to cancer type, tumour microenvironment and hormonal status [56,70]. These versatile bioactive compounds from pomegranates hold significant therapeutic potential due to their diverse pharmacological activities. Punicalagin and ellagic acid have demonstrated the ability to modulate angiogenesis by downregulating the expression of VEGF and VEGF-like growth factors and MMPs, thereby inhibiting blood vessel formation [71].

### 4.2. Flavonoids

Flavonoids, abundant in vegetables, fruits and certain beverages, serve as potent secondary metabolites with diverse health benefits. They exhibit antioxidant, anti-inflammatory, neuroprotective, cardioprotective, antiviral and antibacterial properties [72]. Notably, flavonoids are under investigation for their potential in cancer prevention by targeting endothelial cells and modulating angiogenesis. Indeed, several flavonoids have demonstrated efficacy in inhibiting angiogenesis and metastasis by regulating multiple signalling pathways. They affect the expression of key factors such as VEGF, bFGF, HIF-1α, MMPs and VEGFR, while also inhibiting NF-κB, PI3K/AKT/mTOR and MAPK/ERK signalling pathways. Additionally, flavonoids play a role in adjusting endothelial cell proliferation and migration [73].

Among flavonoids, anthocyanins—a subclass particularly abundant in pomegranate fruits—show multiple anti-carcinogenic effects. Anthocyanins, a subclass of flavonoids, have demonstrated multiple anti-carcinogenic effects. These include direct scavenging of ROS, stimulation of Phase II detoxification enzymes, and reduction of oxidative DNA damage. Specific anthocyanins like cyanidin, delphinidin and malvidin act through the Keap1/Nrf2 pathway, inhibiting caspase-3 via glutathione-related enzymes [74]. Delphinidin, for instance, blocks VEGF-induced phosphorylation in endothelial cells by modulating MAPK/ERK and PI3K/AKT/mTOR pathways [75].

Kaempferol and quercetin, both flavonols present in pomegranate, exhibit significant anti-cancer properties. Kaempferol disrupts cancer cell VEGF release and reduces VEGF-stimulated cell viability by downregulating PI3K/AKT/mTOR and MAPK/ERK pathways [76]. Furthermore, kaempferol potentiates phosphorylation of endothelial Nitric Oxide Synthase (eNOS) and VEGFR-2 in endothelial cells [77]. Quercetin inhibits angiogenesis in human retinal endothelial cells by targeting the VEGFR2, MAPK/ERK, PI3K/AKT/mTOR and MAPK/JNK pathways [78]. Lastly, luteolin suppresses VEGF expression via HIF-1α-dependent mechanisms and inhibits ROS production [79]. It also reduces MMP-1 and MMP-9 expression, contributing to decreased angiogenesis in gastric cancer [80,81].

Pomegranate-derived oestrogenic flavonoids, including luteolin, quercetin and kaempferol, have been suggested to inhibit angiogenesis through multiple mechanisms. These include downregulating the expression of angiogenic growth factors, disrupting key cellular signalling pathways like PI3K/AKT/mTOR, and exerting antioxidant effects that reduce oxidative stress [82].

### 4.3. Fatty Acids and Their Derivatives

Dietary fatty acids play a crucial role in modulating vascular function, particularly through their impact on endothelial cells. Polyunsaturated fatty acids (PUFAs), especially n-3, have been shown to regulate endothelial function effectively. High-fat diets rich in n-6 fatty acids are associated with poor prognosis in breast cancer patients. In a nude mouse model, such diets enhanced breast cancer progression, whereas n-3 fatty acids exhibited suppressive effects associated with impaired angiogenesis. Lipoxygenase and cyclooxygenase products of n-6 fatty acid metabolism have been identified as angiogenic in in vitro assays. Interestingly, dietary saturated and trans fats (excluding cholesterol) can induce hepatic angiogenesis and lymph angiogenesis, promoting hepatic tumours [83].

Punicic acid (C18:3, n-5), a conjugate of linolenic acid, is a notable compound of pomegranate consisting of 18 carbon atoms with three double bonds located at positions 9, 11 and 13. It constitutes approximately 70% of the fatty acids in pomegranate kernel oil [48]. Punicic acid exhibits significant anti-proliferative, pro-apoptotic, anti-inflammatory and antiangiogenic properties. Specifically, these effects have been observed against breast cancer cells MCF7 and MDA-MB-231, prostate cancer cells PC-3, lung non-small cells A549, ovarian cells SKOV3, and glioblastoma T98 GBM [29,84,85]. The anti-inflammatory properties are evident against TNF-α-induced neutrophils and LPS-stimulated Caco-2 cells, where punicic acid inhibits ROS production by targeting the p38 MAPK and Ser345-p47phox axis [86,87].

Punicic acid has also shown significant neuroprotective effects [88]. Acting as a potent anti-inflammatory and antioxidant agent, it modulates neuronal function by inhibiting NF-κB activation and reducing the release of inflammatory cytokines like TNF-α. This reduction in cytokines decreases neuroinflammation, tau hyperphosphorylation, and Aβ formation and aggregation [89]. Additionally, punicic acid inhibits calpain and CDK5 activation, further limiting tau hyperphosphorylation. It enhances GLUT4 protein expression, improving brain glucose metabolism and reducing insulin resistance. Its antioxidative properties boost the PON1 complex, reducing ROS generation and mitigating mitochondrial dysfunction and neuronal apoptosis. Punicic acid also alters HDL lipid composition, reducing oxysterol formation and increasing oxidative resistance, thereby decreasing Aβ plaque formation. Despite these promising effects, research on punicic acid in neuronal cultures is limited, with in vivo studies dating back over a decade [90]. More recent and comprehensive studies are needed to fully elucidate its potential benefits in neuroprotection and cancer therapy.

Lipoic acid, a sulphur-containing compound with potent antioxidant properties derived from caprylic acid, plays a pivotal role in regulating angiogenesis. While its presence has been reported in pomegranate juice [91], conclusive evidence regarding its significant presence or quantification in various parts of the fruit or its by-products remains lacking and requires further investigation. Pomegranate is not a primary source of lipoic acid, but it is more abundantly found in foods such as spinach, broccoli, and organ meats like liver and kidneys. Nevertheless, lipoic acid merits inclusion in this review not only for completeness but also due to its well-documented health-promoting properties and its relevance to angiogenesis and related physiological processes. Lipoic acid neutralises ROS and regenerates key antioxidants, including glutathione and vitamin C, thereby stabilising the cellular microenvironment and influencing angiogenic signalling pathways. Studies emphasise its role in modulating extracellular matrix and angiogenesis-related gene expression, particularly in wound healing under hyperbaric oxygen therapy, where it reduces inflammatory cytokines and regulates VEGF and MMP activity [92].

Beyond its angiogenic properties, lipoic acid exhibits broad biological activity, functioning in both intracellular and extracellular environments. It has demonstrated promise in treating conditions such as diabetic neuropathy and cancer, where it inhibits cancer cell proliferation and modulates tumour suppressor genes. Notably, research suggests that lipoic acid enhances the efficacy of chemotherapeutic agents like paclitaxel—a widely used microtubule-stabilising drug in cancer therapy—through synergistic effects [93].

With its anti-inflammatory, redox-modulating and multifaceted biological properties, lipoic acid presents significant potential for promoting angiogenesis and addressing a range of clinical challenges.

### 4.4. Polysaccharides

Polysaccharides, one of life’s fundamental substances, exhibit a wide array of biological activities, including antioxidative, immunomodulatory, antitumour, anti-inflammatory and hypoglycaemic properties. Their antitumour mechanisms are multifaceted, involving cell cycle arrest, antiangiogenesis, apoptosis and immunomodulation, all of which are closely related to their structure and bioavailability [94]. Clinically, polysaccharides extracted from mushrooms such as *Lentinula edodes* (Berk.) Pegler (lentinan), *Polyporus umbellatus* (Pers.) Fr., *Ganoderma lucidum* (Curtis) P. Karst., and from the genus of Fabaceae *Astragalus* have been employed as antitumour drugs or adjuvants. These compounds either directly target tumour cells or enhance the body’s immune response, demonstrating significant therapeutic efficacy in cancer treatment [95].

Notably, polysaccharide extracts from various plants have shown pronounced antiangiogenic effects. For instance, water-soluble polysaccharides extracted from the roots of *Polygala tenuifolia* Willd. significantly downregulated the protein and mRNA levels of Epidermal Growth Factor Receptor (EGFR), VEGF and the Cluster of Differentiation 34 (CD34) transmembrane cell-cell adhesion factor in tumour-bearing BALB/c mice, thereby inhibiting tumour growth [96]. Similarly, Ren et al. [97] reported that dandelion polysaccharide exhibits antiangiogenic properties, evidenced by tube formation and CAM assays. This polysaccharide inhibits VEGF and HIF-1α expression by targeting the PI3K/AKT/mTOR pathway, suggesting a novel strategy for tumour growth suppression. A homogeneous heteropolysaccharide from *Inula japonica* Thunb. has demonstrated significant antitumour activity in vivo, primarily through immune system activation and angiogenesis inhibition rather than direct cytotoxicity. This mechanism involves interactions with key proteins such as TLR4, programmed death receptor-1 (PD-1) and VEGF [95].

Partially purified pectin fractions from pomegranate rind have shown a dose-dependent inhibition of blood vessel formation in chicken embryos, indicating their potential in antiangiogenic therapy. Although the exact mechanisms are yet to be fully elucidated, it is hypothesised that pectins may mitigate inflammation, disrupt growth factor activity, or affect signalling pathways regulating angiogenesis [98]. Furthermore, galactomannan isolated from pomegranate rind has been recognised for its robust antioxidant, immunomodulatory and anticancer properties in both in vitro and in vivo models. This polysaccharide significantly reduces neovascularization in chick embryos and impairs the invasion, migration and clonogenic capacity of both human and murine cancer cells. Molecular analysis revealed that galactomannan downregulates key pro-angiogenic and pro-metastatic factors such as VEGF, MMP-2 and MMP-9 while upregulating the tissue inhibitors of metalloproteinases TIMP-1 and TIMP-2. Its anti-metastatic efficacy was further validated in a pulmonary metastasis C57BL/6 mice model, where combined treatment with vincristine enhanced survival rates and reduced metastatic indices. Additionally, galactomannan’s inhibition of angiogenesis was confirmed through a peritoneal angiogenesis assay in a BALB/c mice ascitic tumour model [99].

## 5. The Implications of the Antiangiogenic Effect of Pomegranate Extracts

In recent years, the study of phytocomplexes has progressed significantly, driven by advances in omics disciplines and systems biology. Phytocomplexes—mixtures of active and inactive molecules derived from plant extracts—present distinct advantages over isolated single molecules, often leading to enhanced overall health-promoting efficacy. These benefits arise from the synergistic interactions between the various constituents of phytocomplexes and their molecular targets. By leveraging these combined effects, treatments can be tailored to the individual’s specific needs. This personalised approach allows for more effective therapeutic strategies, as it considers the complex interactions within the phytocomplex and their relevance to the patient’s unique biological makeup. Consequently, whole pomegranate extracts have attracted attention for their diverse bioactive properties, particularly their antiangiogenic effects.

**Figure 6:**
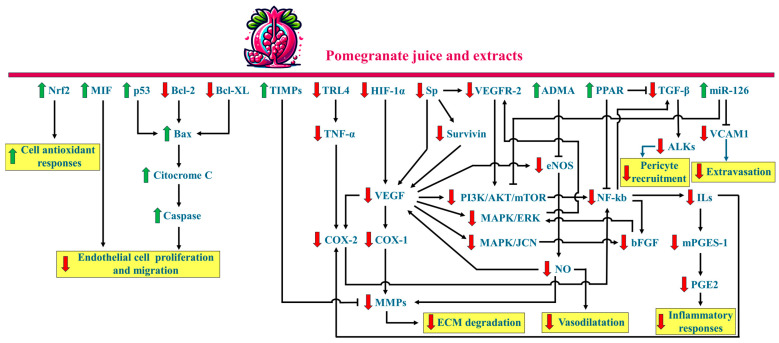
Schematic illustration of the primary molecular targets of pomegranate juice and extracts in relation to angiogenesis and their interactions. ADMA, Asymmetric dimethylarginine; ALKs, Activin receptor-like kinases; AKT, Protein Kinase B; Bax, Bcl-2-associated X protein; Bcl-2, B-cell lymphoma-2; Bcl-XL, B-cell lymphoma-extra-large; bFGF, basic Fibroblast Growth Factor; COX1, Cyclooxygenase 1; COX2; Cyclooxygenase 2; eNOS, endothelial Nitric Oxide Synthase; ERK, Extracellular Signal-Regulated Kinase; HIF-1α, Hypoxia Inducible Factor 1-alpha; ILs, Interleukins; JNK, c-Jun *N*-terminal kinase; MAPK, Mitogen-Activated Protein Kinase; MIF, Macrophage Migration Inhibitory Factor; miR126, MicroRNA 126; MMPs, Matrix metalloproteinases; mPGES-1, Microsomal Prostaglandin E Synthase-1; mTOR, mechanistic Target of Rapamycin; NF-kB, Nuclear Factor kappa-light-chain-enhancer of activated B cells; Nrf2, Nuclear factor erythroid 2-related factor 2; NO, Nitric oxide; p53, protein 53; PI3K, Phosphoinositide 3-kinase; PGE2, Prostaglandin E 2; PPAR, Peroxisome Proliferator-Activated Receptor; Sp, Specificity proteins; TGF-β, Transforming Growth Factor beta; TIMPs, tissue inhibitors of metalloproteinases; TNFα, Tumor Necrosis Factor alpha; TRL4, Toll-like receptor-4; VEGF, Vascular Endothelial Growth Factor; VCAM1, Vascular Cell Adhesion Molecule 1; VEGFR-2, Vascular Endothelial Growth Factor Receptor-2.

The antiangiogenic effect of pomegranate stems from its ability to modulate the expression and activity of key angiogenic factors, resulting in a complex interplay between pro-angiogenic and antiangiogenic molecules (Figure 6). This effect is observed across multiple cancer types, including prostate, skin, breast, pancreatic and colon cancers [100]. For instance, a powdered pomegranate extract rich in ellagitannins—derived from peels and standardised to contain 37% ellagitannins (as punicalagin) and 3.5% free ellagic acid—significantly inhibited proliferation and reduced HIF-1α and VEGF levels in prostate cancer cells (LNCaP) and HUVECs under hypoxic conditions in vitro. This extract also decreased tumour size, vessel density, and the expression of HIF-1α and VEGF in prostate cancer xenografts in severe combined immunodeficient (SCID) mice, indicating its potential to inhibit tumour growth and angiogenesis in vivo [101]. Similarly, studies on B16F10 melanoma and HUVECs have shown that ethanolic pomegranate peel extracts inhibit malignant cell proliferation and angiogenesis. In HUVECs, the extract significantly reduced tube formation and VEGF mRNA expression. These effects were reversed by PPAR antagonists, suggesting that the antiangiogenic effects of the extract may be mediated through a PPAR-dependent pathway [102,103]. Pomegranate extracts significantly reduced tumour weight and haemoglobin content in human pancreatic cancer (Suit-2) and colon cancer (Colo205) cell lines. The extract also showed promise in treating benign prostatic hypertrophy (BPH) reducing the proliferation and migration rates of BPH-1 cells by up to 60%, along with a decrease in the release of inflammatory cytokines such as IL-6 and PGE2, as well as pro-angiogenic factors like VEGF. Pomegranate extracts also elevated the levels of Asymmetric Dimethylarginine (ADMA), an endogenous inhibitor of eNOS that functions as a negative regulator of angiogenesis. Furthermore, pomegranate extract reduced angiogenesis in vitro by inhibiting tube formation in human endothelial cells, suggesting its potential as a therapeutic option to prevent neovascularization in BPH and its progression to prostate cancer [104].

In a DMBA-induced rat breast cancer model, whole pomegranate extract, either alone or in combination with tangeretin—an *O*-polymethoxylated flavone from citrus peels—, significantly decreased plasma VEGF levels, while increasing p53 and Bax expressions, indicating a potential role in breast cancer prevention [105]. In the same in vivo system, a pomegranate emulsion composed of seed oil in aqueous pomegranate extract suppressed the expression of inflammatory mediators, including iNOS, COX-2, prostaglandin E2 (PGE-2) and HSP90 [106,107]. Moreover, treatment with methanolic pomegranate peel extract in triple-negative breast cancer cells resulted in marked inhibition of cell migration and alterations in gene expression related to cancer cell migration, including downregulation of VEGF [108].

In AOM-treated rats, pomegranate juice also demonstrated significant effects downregulating the expression of iNOS, COX-2, NF-κB and VCAM-1, inhibiting the phosphorylation of PI3K/AKT and mTOR, and increasing the expression of miR-126. This micro–RNA is abundant in endothelial cells and progenitor cells, where it regulates angiogenesis by promoting VEGF signalling and maintaining vascular integrity, as well as attenuating inflammatory responses [109]. Additionally, pomegranate juice significantly reduced the level of secreted IL-6, IL-12p40, IL-1β and CCL5 (C-C motif chemokine ligand 5) in prostate cancer cells [110].

An earlier study in 2003 found that the antiangiogenic effects of pomegranate supercritical CO_2_-extracted seed oil and fermented juice polyphenols involved downregulation of VEGF in some cancer cell types (MCF-7) and upregulation of the angiogenic suppressor Macrophage Migration Inhibitory Factor (MIF) in others (MDA-MB-231). These extracts also reduced tube formation in HUVECs and decreased new blood vessel formation in the CAM model. In contrast, cold-pressed pomegranate seed oil and pomegranate peel extract had only mild effects on VEGF levels. The greater efficacy of polyphenols in fermented juice, compared to those from the peels, has been attributed to the breakage of flavonoid-sugar complexes during fermentation, a process akin to stomach hydrolysis [111]. Pomegranate fractions, particularly seed oil, also exhibit oestrogenic activity, influencing vascular endothelium through oestrogen receptors and NF-kB pathways. In a study involving MCF-7 breast cancer cells co-cultured with adipose-derived stem cells (ADSCs), pomegranate seed extract enhanced the inhibitory effects of ADSCs on cancer cell viability and proliferation, increased apoptosis, and downregulated VEGF gene expression [30].

Moga et al. [112] reported that the antiangiogenic potential of pomegranate extracts also involves the downregulation of Specificity proteins (Sp) transcription factors, which regulate the expression of VEGF, VEGFR and survivin. Survivin, a member of the inhibitor of apoptosis (IAP) family, is highly expressed in most human tumours and foetal tissues and is capable of independently promoting VEGF expression.

Other parts of the pomegranate have also demonstrated significant antiangiogenic and vasoprotective effects. For example, an ethanolic extract of pomegranate root inhibited angiogenesis in a CAM assay and protected against glucose-induced vascular changes. These effects were attributed to punicalin, punicalagin and ellagic acid, compounds present also in the roots, through antioxidant mechanisms and the inhibition of VEGF and VEGF-like growth factors [113].

## 6. Conclusions

Extensive evidence underscores the significant antiangiogenic potential of pomegranate extracts, positioning them as promising candidates for the prevention and treatment of cancer and other angiogenesis-related diseases. This potential is primarily attributed to their rich content of polyphenols, punicic acid and bioactive polysaccharides, which modulate key molecular targets such as HIF-1α, VEGF, Sp, specific microRNAs and various inflammatory mediators. Beyond their antiangiogenic properties, the diverse bioactive compounds found in various parts of the pomegranate—such as juice, peels, kernels and roots—contribute to a wide range of biological activities, including anti-inflammatory, antioxidant and pro-apoptotic effects. These activities collectively inhibit neovascularization, potentially reducing tumour growth, limiting metastasis, and enhancing the efficacy of conventional cancer therapies.

Moreover, the method of extraction plays a crucial role in determining the efficacy of pomegranate extracts. Certain techniques, particularly green extraction methods, have been shown to enhance the bioavailability and potency of active compounds compared to conventional solvent-based methods. The complex interplay and potential synergistic effects among the various phytochemicals within the extracted phytocomplex may further amplify their therapeutic potential when compared to isolated compounds. Therefore, refining green extraction methods to produce high-quality, non-toxic extracts is essential, especially from a sustainability perspective and in the context of exploiting pomegranate by-products within a circular economy.

Future research should focus on detailed mechanistic studies to deepen our understanding of the precise molecular pathways through which pomegranate extracts exert their antiangiogenic effects. Such insights will be critical for developing targeted therapies that maximise these benefits. Equally important are clinical trials to validate the safety and efficacy of pomegranate extracts in human populations, particularly across different cancer types and other conditions driven by abnormal angiogenesis. Current studies indicate that pomegranate extracts are largely non-cytotoxic when administered topically or orally, suggesting safety for these routes. However, data on systemic toxicity, particularly regarding intravenous use, are still limited. This highlights the need for further research to comprehensively assess the safety profile of intravenous administration of pomegranate extracts. A thorough understanding of potential systemic effects is essential to ensure safe and effective therapeutic applications. Additionally, exploring the potential synergistic effects of combining pomegranate extracts with other natural or synthetic agents could lead to the development of more effective combination therapies.

Advances in biotechnology and nanotechnology offer promising avenues for improving the delivery, stability and bioavailability of pomegranate extracts, ensuring their full therapeutic potential is realised. Additionally, progress in nutrigenomics provides new insights into how pomegranate extracts could be tailored to individual genetic profiles, enhancing their effectiveness in managing angiogenesis-related diseases. Given their accessibility, low cost and minimal side effects, pomegranate extracts represent a valuable option for addressing these conditions. Continued research and innovation in this area will be crucial for integrating pomegranate extracts into personalised medicine and broader therapeutic strategies, potentially transforming the landscape of treatment for these conditions.

## List of Abbreviations

ADMA, Asymmetric dimethylarginine; ADSCs, Adipose-Derived Stem Cells; AKT, Protein Kinase B; ALKs, Activin receptor-like kinases; AP-1, Activator Protein-1; Aβ42, amyloid beta 42 peptide; Bad, Bcl-2-associated death promoter; Bak, Bcl-2 homologous antagonist/killer; Bax, Bcl-2-associated X protein; Bcl-2, B-cell lymphoma 2 protein; Bcl-XL, B-cell lymphoma-extra-large; bFGF, basic Fibroblast Growth Factor; BM, basement membrane; BPH, Benign Prostatic Hypertrophy; CAM, chick embryo chorioallantoic membrane; CCL5, C-C motif chemokine ligand 5; CD34, Cluster of Differentiation 34; CDH5, Cadherin 5; CDK, Cyclin-dependent kinase; CDKNs, Cyclin-dependent kinase inhibitors; COX1/2, Cyclooxygenase 1/2; CXCL1/8, C-X-C ligand-1/8; CXCR2/4, C-X-C chemokine receptor 2/4; DLL4, Delta-like ligand 4; DMBA, 7,12-dimethylbenzanthracene; E-caderin, Epitelial caderin; ECs, endothelial cells; EGFR, Epidermal Growth Factor Receptor; eNOS, endothelial Nitric Oxide Synthase; ERK, Extracellular Signal-Regulated Kinase; GCLC, Glutamate-Cysteine Ligase Catalytic Subunit; GCLM, Glutamate-Cysteine Ligase Modifier Subunit; GLUT4, Glucose Transporter Type 4; GSTA4, Glutathione S-Transferase Alpha 4; GSTM1, Glutathione S-Transferase Mu 1; HDL, High-Density Lipoprotein; HIFs, Hypoxia-Inducible Factors; HMMR, Hyaluronan-Mediated Motility Receptor; HO-1, Heme Oxygenase 1; HSP90, Heat Shock Protein 90; HUVECs, Human Umbilical Vein Endothelial Cells; IAP, inhibitor of apoptosis; ICAMs, Intercellular Adhesion Molecules; ILs, Interleukins; iNOS, inducible Nitric Oxide Synthase; JNK, c-Jun N-terminal kinase; JunB, Jun B proto-oncogene; Keap1, Kelch-like ECH-associated protein 1; KIP1/P27, Cyclin-dependent kinase inhibitor 1B; LDL, Low-Density Lipoprotein; MAPK, Mitogen-Activated Protein Kinase; MGO, methylglyoxal; MIF, Macrophage Migration Inhibitory Factor; miRs, microRNAs; MMPs, Matrix metalloproteinases; mPGES-1, Microsomal Prostaglandin E Synthase-1; mTOR, mechanistic Target of Rapamycin; NF-kB, Nuclear Factor kappa-light-chain-enhancer of activated B cells; NO, Nitric Oxide; NQO-1, NAD(P)H Quinone Dehydrogenase 1; Nrf2, Nuclear factor erythroid 2-related factor 2; p47phox, phagocyte oxidase 47 kDa; p53, protein 53; PD-1, Programmed Death receptor-1; PDGFs, Platelet-Derived Growth Factors; PECAM1, Platelet Endothelial Cell Adhesion Molecule; PGC-1α, Peroxisome proliferator-activated receptor gamma coactivator-1 alpha; PGE2, Prostaglandin E 2; PI3K, Phosphoinositide 3-kinase; PON1, Paraoxonase 1; PPARs, Peroxisome Proliferator-Activated Receptors; PUFAs, Polyunsaturated fatty acids; ROS, Reactive Oxygen Species; SCID, severe combined immunodeficient; SIRT1, Sirtuin 1; SOD, Superoxide Dismutase; Sp, Specificity proteins; TGF-β, Transforming Growth Factor beta; TIMPs, tissue inhibitors of metalloproteinases; TNF-α, Tumor Necrosis Factor alpha; TRL4, Toll-like receptor-4; UPAs, Urokinase Plasminogen Activators; VCAMs, Vascular Cell Adhesion Molecules; VEGFR-2, Vascular Endothelial Growth Factor Receptor-2; VEGFs, Vascular Endothelial Growth Factors.

## References

[1] Dudley A.C., Griffioen A.W. (2023). Pathological angiogenesis: Mechanisms and therapeutic strategies. Angiogenesis.

[2] Patel S.A., Nilsson M.B., Le X., Cascone T., Jain R.K., Heymach J.V. (2023). Molecular Mechanisms and Future Implications of VEGF/VEGFR in Cancer Therapy. Clin. Cancer Res..

[3] Davidescu L., Precup A.I., Fodor R., Ilias T.I. (2024). Investigating Mechanisms and Causes Related to Angiogenesis: A Review. Arch. Pharm. Pract..

[4] Karl E., Zhang Z., Dong Z., Neiva K.G., Soengas M.S., Koch A.E., Polverini P.J., Núñez G., Nör J.E. (2007). Unidirectional crosstalk between Bcl-x_L_ and Bcl-2 enhances the angiogenic phenotype of endothelial cells. Cell Death Differ..

[5] Schmidt D., Textor B., Pein O.T., Licht A.H., Andrecht S., Sator-Schmitt M., Fusenig N.E., Angel P., Schorpp-Kistner M. (2007). Critical role for NF-κB-induced JunB in VEGF regulation and tumor angiogenesis. EMBO J..

[6] Francavilla C., Maddaluno L., Cavallaro U. (2009). The functional role of cell adhesion molecules in tumor angiogenesis. Semin. Cancer Biol..

[7] Quintero-Fabián S., Arreola R., Becerril-Villanueva E., Torres-Romero J.C., Arana-Argáez V., Lara-Riegos J., Ramírez-Camacho M.A., Alvarez-Sánchez M.E. (2019). Role of Matrix Metalloproteinases in Angiogenesis and Cancer. Front. Oncol..

[8] Liu B., Yang H., Song Y.S., Sorenson C.M., Sheibani N. (2024). Thrombospondin-1 in vascular development, vascular function, and vascular disease. Semin. Cell Dev. Biol..

[9] Lauer G., Sollberg S., Cole M., Flamme I., Stürzebecher J., Mann K., Krieg T., Eming S.A. (2000). Expression and proteolysis of vascular endothelial growth factor is increased in chronic wounds. J. Investig. Dermatol..

[10] Aguilar-Cazares D., Chavez-Dominguez R., Carlos-Reyes A., Lopez-Camarillo C., Hernadez de la Cruz O.N., Lopez-Gonzalez J.S. (2019). Contribution of Angiogenesis to Inflammation and Cancer. Front. Oncol..

[11] Albini A., Noonan D.M., Corradino P., Magnoni F., Corso G. (2024). The Past and Future of Angiogenesis as a Target for Cancer Therapy and Prevention. Cancer Prev. Res..

[12] Liu Z.L., Chen H.H., Zheng L.L., Sun L.P., Shi L. (2023). Angiogenic signaling pathways and anti-angiogenic therapy for cancer. Sig. Transduct. Target. Ther..

[13] Chaudhary P., Janmeda P., Docea A.O., Yeskaliyeva B., Abdull Razis A.F., Modu B., Calina D., Sharifi-Rad J. (2023). Oxidative stress, free radicals and antioxidants: Potential crosstalk in the pathophysiology of human diseases. Front. Chem..

[14] Ribatti D. (2008). Judah Folkman, a pioneer in the study of angiogenesis. Angiogenesis.

[15] Quesada A.R., Muñoz-Chápuli R., Medina M.A. (2006). Anti-angiogenic drugs: From bench to clinical trials. Med. Res. Rev..

[16] Lopes-Coelho F., Martins F., Pereira S.A., Serpa J. (2021). Anti-Angiogenic Therapy: Current Challenges and Future Perspectives. Int. J. Mol. Sci..

[17] Haibe Y., Kreidieh M., El Hajj H., Khalifeh I., Mukherji D., Temraz S., Shamseddine A. (2020). Resistance Mechanisms to Anti-angiogenic Therapies in Cancer. Front. Oncol..

[18] Jiang X., Wang J., Deng X., Xiong F., Zhang S., Gong Z., Li X., Cao K., Deng H., He Y. (2020). The role of microenvironment in tumor angiogenesis. J. Exp. Clin. Cancer Res..

[19] Furukawa K., Nagano T., Tachihara M., Yamamoto M., Nishimura Y. (2020). Interaction between Immunotherapy and Antiangiogenic Therapy for Cancer. Molecules.

[20] Yücel E.I., Sahin M. (2020). Fenretinide reduces angiogenesis by downregulating CDH5, FOXM1 and eNOS genes and suppressing microRNA-10b. Mol. Biol. Rep..

[21] Christyraj J.D.S., Rajagopalan K., Kadalpandian P., Raja L.B., Radhakrishnan N., Vasantha S., Pandurangan A.K. (2023). Anti-angiogenic activities of natural compounds from plant sources. Natural Products as Cancer Therapeutics.

[22] Li R., Song X., Guo Y., Song P., Duan D., Chen Z.S. (2021). Natural Products: A Promising Therapeutics for Targeting Tumor Angiogenesis. Front. Oncol..

[23] Malabadi R.B., Sadiya M.R., Kolkar K.P., Mammadova S.S., Chalannavar R.K., Baijnath H. (2024). Role of Plant derived-medicine for controlling Cancer. Int. J. Sci. Res. Arch..

[24] Rahimi H.R., Arastoo M., Ostad S.N. (2012). A Comprehensive Review of *Punica granatum* (Pomegranate) Properties in Toxicological, Pharmacological, Cellular and Molecular Biology Researches. Iran. J. Pharm. Res..

[25] Khan G.J., Omer M.O., Ashraf M., Rehman H.U., Khan Z.U.D. (2013). Effect of *Punica granatum* (pomegranate) fruit extract on angiogenesis. J. App. Pharm..

[26] Sudha T., Mousa D.S., El-Far A.H., Mousa S.A. (2021). Pomegranate (*Punica granatum*) Fruit Extract Suppresses Cancer Progression and Tumor Angiogenesis of Pancreatic and Colon Cancer in Chick Chorioallantoic Membrane Model. Nutr. Cancer.

[27] Rahmani A.H., Alsahli M.A., Almatroodi S.A. (2017). Active Constituents of Pomegranates (*Punica granatum*) as Potential Candidates in the Management of Health through Modulation of Biological Activities. Pharmacogn. J..

[28] Bassiri-Jahromi S. (2018). *Punica granatum* (Pomegranate) activity in health promotion and cancer prevention. Oncol. Rev..

[29] Costantini S., Rusolo F., De Vito V., Moccia S., Picariello G., Capone F., Guerriero E., Castello G., Volpe M.G. (2014). Potential anti-inflammatory effects of the hydrophilic fraction of pomegranate (*Punica granatum* L.) seed oil on breast cancer cell lines. Molecules.

[30] Moradi-Gharibvand N., Setayeshmehr M., Kazemi M., Safaee A., Khorsandi L.S., Nejad D.B., Hasheminia S.J., Hashemibeni B. (2022). Pomegranate seed extract enhances the inhibitory effect of adipose-derived mesenchymal stem cells on breast cancer cell line in co-culture conditions. Res. Pharm. Sci..

[31] Zarfeshany A., Asgary S., Javanmard S.H. (2014). Potent health effects of pomegranate. Adv. Biomed. Res..

[32] Montefusco A., Durante M., Migoni D., De Caroli M., Ilahy R., Pék Z., Helyes L., Fanizzi F.P., Mita G., Piro G. (2021). Analysis of the Phytochemical Composition of Pomegranate Fruit Juices, Peels and Kernels: A Comparative Study on Four Cultivars Grown in Southern Italy. Plants.

[33] Esposto S., Veneziani G., Taticchi A., Urbani S., Selvaggini R., Sordini B., Daidone L., Gironi G., Servili M. (2021). Chemical Composition, Antioxidant Activity, and Sensory Characterization of Commercial Pomegranate Juices. Antioxidants.

[34] Azmat F., Safdar M., Ahmad H., Khan M.R.J., Abid J., Naseer M.S., Aggarwal S., Imran A., Khalid U., Zahra S.M. (2024). Phytochemical profile, nutritional composition of pomegranate peel and peel extract as a potential source of nutraceutical: A comprehensive review. Food Sci. Nutr..

[35] Khadivi A., Rezagholi M., Shams M. (2024). Phytochemical properties and bioactive compounds of pomegranate (*Punica granatum* L.). Hortic. Sci. Biotechnol.

[36] Pirzadeh M., Caporaso N., Rauf A., Shariati M.A., Yessimbekov Z., Khan M.U., Imran M., Mubarak M.S. (2021). Pomegranate as a source of bioactive constituents: A review on their characterization, properties and applications. Crit. Rev. Food Sci. Nutr..

[37] Fernandes L., Pereira J.A., Lopez-Cortes I., Salazar D.M., Ramalhosa E., Casal S. (2015). Lipid composition of seed oils of different pomegranate (*Punica granatum* L.) cultivars from Spain. Int. J. Food Stud..

[38] Ceci C., Lacal P.M., Tentori L., De Martino M.G., Miano R., Graziani G. (2018). Experimental Evidence of the Antitumor, Antimetastatic and Antiangiogenic Activity of Ellagic Acid. Nutrients.

[39] Maphetu N., Unuofin J.O., Masuku N.P., Olisah C., Lebelo S.L. (2022). Medicinal uses, pharmacological activities, phytochemistry, and the molecular mechanisms of *Punica granatum* L. (pomegranate) plant extracts: A review. Biomed. Pharmacother..

[40] Fourati M., Smaoui S., Hlima H.B., Elhadef K., Braïek O.B., Ennouri K., Mtibaa A.C., Mellouli L. (2020). Bioactive Compounds and Pharmacological Potential of Pomegranate (*Punica granatum*) Seeds—A Review. Plant Foods Hum. Nutr..

[41] Morvaridzadeh M., Sepidarkish M., Daneshzad E., Akbari A., Mobini G.R., Heshmati J. (2020). The effect of pomegranate on oxidative stress parameters: A systematic review and meta-analysis. Complement. Ther. Med..

[42] Alami M., Boumezough K., Zerif E., Zoubdane N., Khalil A., Bunt T., Laurent B., Witkowski J.M., Ramassamyand C., Boulbaroud S. (2024). Pomegranate (*Punica granatum* L.) Bioactive Compounds Alleviate Human Beta-Amyloid-(1-42)-Induced Tau-phosphorylation, Neuronal Death, Oxidative Stress and Reduced LPS-Induced Neuroinflammation in Alzheimer’s Disease. Med. Pharmacol..

[43] Shin Y.J., Evitts K.M., Jin S., Howard C., Sharp-Milgrom M., Schwarze-Taufiq T., Kinoshita C., Young J.E., Zheng Y. (2023). Amyloid beta peptides (Aβ) from Alzheimer’s disease neuronal secretome induce endothelial activation in a human cerebral microvessel model. Neurobiol. Dis..

[44] Steiner K., Humpel C. (2024). Beta-Amyloid Enhances Vessel Formation in Organotypic Brain Slices Connected to Microcontact Prints. Biomolecules.

[45] Benedetti G., Zabini F., Tagliavento L., Meneguzzo F., Calderone V., Testai L. (2023). An Overview of the Health Benefits, Extraction Methods and Improving the Properties of Pomegranate. Antioxidants.

[46] Cano-Lamadrid M., Martínez-Zamora L., Castillejo N., Artés-Hernández F. (2022). From Pomegranate Byproducts Waste to Worth: A Review of Extraction Techniques and Potential Applications for Their Revalorization. Foods.

[47] Kupnik K., Leitgeb M., Primožič M., Postružnik V., Kotnik P., Kučuk N., Knez Ž., Marevci M.K. (2022). Supercritical Fluid and Conventional Extractions of High Value-Added Compounds from Pomegranate Peels Waste: Production, Quantification and Antimicrobial Activity of Bioactive Constituents. Plants.

[48] Durante M., Montefusco A., Marrese P.P., Soccio M., Pastore D., Piro G., Mita G., Lenucci M.S. (2017). Seeds of pomegranate, tomato and grapes: An underestimated source of natural bioactive molecules and antioxidants from agri-food by-products. J. Food Compos. Anal..

[49] Faria G.L.M., Silva E.K. (2024). Pulsed electric field, ultrasound and microwave heating based extraction techniques for valorization of pomegranate peel by-products: A review. J. Environ. Chem. Eng..

[50] Pathak P.D., Mandavgane S.A., Kulkarni B.D. (2017). Valorization of Pomegranate Peels: A Biorefinery Approach. Waste Biomass Valor..

[51] Bañares C., Chabni A., de Donlebún B.P., Reglero G., Torres C.F. (2023). Chemical characterization of pomegranate and alfalfa seed oils obtained by a two-step sequential extraction procedure of expeller and supercritical CO_2_ technologies. J. Food Compos. Anal..

[52] Talekar S., Patti A.F., Singh R., Vijayraghavan R., Arora A. (2018). From waste to wealth: High recovery of nutraceuticals from pomegranate seed waste using a green extraction process. Ind. Crop. Prod..

[53] Shinde P.N., Mandavgane S.A., Karadbhajane V. (2020). Process development and life cycle assessment of pomegranate biorefinery. Environ. Sci. Pollut. Res..

[54] Guzmán-Lorite M., Marina M.L., García M.C. (2022). Successive extraction using natural deep eutectic solvents and pressurized liquids for a greener and holistic recovery of proteins from pomegranate seeds. Food Res. Int..

[55] Siddiqui S.A., Singh S., Nayik G.A. (2024). Bioactive compounds from pomegranate peels—Biological properties, structure–function relationships, health benefits and food applications—A comprehensive review. J. Funct. Foods.

[56] Larrosa M., García-Conesa M.T., Espín J.C., Tomás-Barberán F.A. (2010). Ellagitannins, ellagic acid and vascular health. Mol. Aspects Med..

[57] Feng L., Yin Y., Fang Y., Yang X. (2017). Quantitative determination of punicalagin and related substances in different parts of pomegranate. Food Anal. Methods.

[58] Liu W., Ou Y., Yang Y., Zhang X., Huang L., Wang X., Wu B., Huang M. (2021). Inhibitory Effect of Punicalagin on Inflammatory and Angiogenic Activation of Human Umbilical Vein Endothelial Cells. Front. Pharmacol..

[59] Huang T., Zhang X., Wang H. (2020). Punicalagin inhibited proliferation, invasion and angiogenesis of osteosarcoma through suppression of NF-κB signaling. Mol. Med. Rep..

[60] Berdowska I., Matusiewicz M., Fecka I. (2021). Punicalagin in Cancer Prevention-Via Signaling Pathways Targeting. Nutrients.

[61] Chen P., Guo Z., Lei J., Wang Y. (2024). Pomegranate polyphenol punicalin ameliorates lipopolysaccharide-induced memory impairment, behavioral disorders, oxidative stress, and neuroinflammation via inhibition of TLR4-NF-кB pathway. Phytother. Res..

[62] Ji W., Zhang X., Sang C., Wang H., Zhou K., Zhang Y., Bo L. (2023). Punicalin attenuates LPS-induced acute lung injury by inhibiting inflammatory cytokine production and MAPK/NF-κB signaling in mice. Heliyon.

[63] Huang S.-T., Wang C.-Y., Yang R.-C., Wu H.-T., Yang S.-H., Cheng Y.-C., Pang J.-H.S. (2011). Ellagic acid, the active compound of *Phyllanthus urinaria*, exerts in vivo anti-angiogenic effect and inhibits MMP-2 activity. Evid.-Based Complement. Altern. Med..

[64] Wang N., Wang Z.Y., Mo S.L., Loo T.Y., Wang D.M., Luo H.B., Yang D.P., Chen Y.L., Shen J.G., Chen J.P. (2012). Ellagic acid, a phenolic compound, exerts anti-angiogenesis effects via VEGFR-2 signaling pathway in breast cancer. Breast Cancer Res. Treat..

[65] Ceci C., Tentori L., Atzori M.G., Lacal P.M., Bonanno E., Scimeca M., Cicconi R., Mattei M., De Martino M.G., Vespasiani G. (2016). Ellagic acid inhibits bladder cancer invasiveness and in vivo tumor growth. Nutrients.

[66] Zaazaa A.M., Lokman M.S., Shalby A.B., Ahmed H.H., El-Toumy S.A. (2018). Ellagic Acid Holds Promise Against Hepatocellular Carcinoma in an Experimental Model: Mechanisms of Action. Asian Pac. J. Cancer Prev..

[67] Ghosh N., Das A., Biswas N., Gnyawali S., Singh K., Gorain M., Polcyn C., Khanna S., Roy S., Sen C.K. (2020). Urolithin A augments angiogenic pathways in skeletal muscle by bolstering NAD^+^ and SIRT1. Sci. Rep..

[68] Feng Z.H., Chen J., Yuan P.T., Ji Z.Y., Tao S.Y., Zheng L., Wei X.A., Zheng Z.Y., Zheng B.J., Chen B. (2022). Urolithin A Promotes Angiogenesis and Tissue Regeneration in a Full-Thickness Cutaneous Wound Model. Front. Pharmacol..

[69] Mannino F., Imbesi C., Bitto A., Minutoli L., Squadrito F., D’Angelo T., Booz C., Pallio G., Irrera N. (2023). Anti-oxidant and anti-inflammatory effects of ellagic and punicic acid in an in vitro model of cardiac fibrosis. Biomed. Pharmacother..

[70] Papoutsi Z., Kassi E., Tsiapara A., Fokialakis N., Chrousos G.P., Moutsatsou P. (2005). Evaluation of estrogenic/antiestrogenic activity of ellagic acid via the estrogen receptor subtypes ERalpha and ERbeta. J. Agric. Food Chem..

[71] Vanella L., Di Giacomo C., Acquaviva R., Barbagallo I., Li Volti G., Cardile V., Abraham N.G., Sorrenti V. (2013). Effects of Ellagic Acid on Angiogenic Factors in Prostate Cancer Cells. Cancers.

[72] Fan X., Fan Z., Yang Z., Huang T., Tong Y., Yang D., Mao X., Yang M. (2022). Flavonoids—Natural Gifts to Promote Health and Longevity. Int. J. Mol. Sci..

[73] Subbaraj G.K., Kumar Y.S., Kulanthaivel L. (2021). Antiangiogenic role of natural flavonoids and their molecular mechanism: An update. Egypt. J. Intern. Med..

[74] Shih P.H., Yeh C.T., Yen G.C. (2007). Anthocyanins Induce the Activation of Phase II Enzymes through the Antioxidant Response Element Pathway against Oxidative Stress-Induced Apoptosis. J. Agric. Food Chem..

[75] Kim Y.E., Hwang C.J., Lee H.P., Kim C.S., Son D.J., Ham Y.W., Hellström M., Han S.B., Kim H.S., Park E.K. (2017). Inhibitory effect of punicalagin on lipopolysaccharide-induced neuroinflammation, oxidative stress and memory impairment via inhibition of nuclear factor-kappaB. Neuropharmacology.

[76] Chin H.K., Horng C.T., Liu Y.S., Lu C.C., Su C.Y., Chen P.S., Chiu H.Y., Tsai F.J., Shieh P.C., Yang J.S. (2018). Kaempferol inhibits angiogenic ability by targeting VEGF receptor-2 and downregulating the PI3K/AKT, MEK and ERK pathways in VEGF-stimulated human umbilical vein endothelial cells. Oncol. Rep..

[77] Hu W.H., Wang H.Y., Xia Y.T., Dai D.K., Xiong Q.P., Dong T.T., Duan R., Chan G.K., Qin Q.W., Tsim K.W. (2020). Kaempferol, a major flavonoid in Ginkgo folium, potentiates angiogenic functions in cultured endothelial cells by binding to vascular endothelial growth factor. Front. Pharmacol..

[78] Lupo G., Cambria M.T., Olivieri M., Rocco C., Caporarello N., Longo A., Zanghì G., Salmeri M., Foti M.C., Anfuso C.D. (2019). Anti-angiogenic effect of quercetin and its 8-methyl pentamethyl ether derivative in human microvascular endothelial cells. J. Cell Mol. Med..

[79] Park S.W., Cho C.S., Jun H.O., Ryu N.H., Kim J.H., Yu Y.S., Kim J.S., Kim J.H. (2012). Anti-angiogenic effect of luteolin on retinal neovascularization via blockade of reactive oxygen species production. Investig. Ophthalmol. Vis. Sci..

[80] Pervin M., Unno K., Nakamura Y., Imai S. (2016). Luteolin Suppresses Ultraviolet A- and B-induced matrix metalloproteinase 1- and 9 expression in human dermal fibroblast cells. J. Nutr. Food Sci..

[81] Zang M., Hu L., Zhang B., Zhu Z., Li J., Zhu Z., Yan M., Liu B. (2017). Luteolin suppresses angiogenesis and vasculogenic mimicry formation through inhibiting Notch1-VEGF signaling in gastric cancer. Biochem. Biophys. Res. Commun..

[82] Wang Y., Huang M., Zhou X., Li H., Ma X., Sun C. (2023). Potential of natural flavonoids to target breast cancer angiogenesis (review). Br. J. Pharmacol..

[83] Rose D.P., Connolly J.M. (2000). Regulation of tumor angiogenesis by dietary fatty acids and eicosanoids. Nutr. Cancer..

[84] Seidi K., Jahanban-Esfahlan R., Abasi M., Abbasi M.M. (2016). Anti tumoral properties of *Punica granatum* (Pomegranate) seed extract in different human cancer cells. Asian Pac. J. Cancer Prev..

[85] Mete M., Unsal U.U., Aydemir I., Sönmez P.K., Tuglu M.I. (2019). Punicic acid inhibits glioblastoma migration and proliferation via the PI3K/AKT1/mTOR signaling pathway. Anti-Cancer Agents Med. Chem..

[86] Boussetta T., Raad H., Lettéron P., Gougerot-Pocidalo M.A., Marie J.C., Driss F., El-Benna J. (2009). Punicic Acid a Conjugated Linolenic Acid Inhibits TNFα-Induced Neutrophil Hyperactivation and Protects from Experimental Colon Inflammation in Rats. PLoS ONE.

[87] Bañares C., Carballeda-Sangiao N., Chabni A., García-Cordero J., Reglero G., de Pascual-Teresa S., Torres C.F. (2023). Anti-inflammatory effect of two pomegranate seed oils obtained by green technologies in Caco-2 cells using the bioaccessible fraction from in vitro gastrointestinal digestion. Food Res. Int..

[88] Guerra-Vázquez C.M., Martínez-Ávila M., Guajardo-Flores D., Antunes-Ricardo M. (2022). Punicic acid and its role in the prevention of neurological disorders: A review. Foods.

[89] Kohno H., Suzuki R., Yasui Y., Hosokawa M., Miyashita K., Tanaka T. (2004). Pomegranate seed oil rich in conjugated linolenic acid suppresses chemically induced colon carcinogenesis in rats. Cancer Sci..

[90] Mizrahi M., Friedman-Levi Y., Larush L., Frid K., Binyamin O., Dori D., Fainstein N., Ovadia H., Ben-Hur T., Magdassi S. (2014). Pomegranate seed oil nanoemulsions for the prevention and treatment of neurodegenerative diseases: The case of genetic CJD. Nanomed. Nanotechnol. Biol. Med..

[91] Viladomiu M., Hontecillas R., Lu P., Bassaganya-Riera J. (2013). Preventive and prophylactic mechanisms of action of pomegranate bioactive constituents. Evid. Based Complement. Alternat. Med..

[92] Alleva R., Tomasetti M., Sartini D., Emanuelli M., Nasole E., Di Donato F., Borghi B., Santarelli L., Neuzil J. (2008). alpha-Lipoic acid modulates extracellular matrix and angiogenesis gene expression in non-healing wounds treated with hyperbaric oxygen therapy. Mol. Med..

[93] Çoban H.Ş., Çil N., Önder E., Abban Mete G. (2024). Anti-cancer effects of alpha lipoic acid, cisplatin and paclitaxel combination in the OVCAR-3 ovarian adenocarcinoma cell line. Mol. Biol. Rep..

[94] Guo R., Chen M., Ding Y., Yang P., Wang M., Zhang H., He Y., Ma H. (2022). Polysaccharides as potential anti-tumor biomacromolecules—A review. Front. Nutr..

[95] Wang X., Li Y., Liu W., Shen Y., Lin Z., Nakajima A., Xu J., Guo Y. (2023). A polysaccharide from Inula japonica showing in vivo antitumor activity by interacting with TLR-4, PD-1, and VEGF. Int. J. Biol. Macromol..

[96] Yao H., Cui P., Xu D., Liu Y., Tian Q., Zhang F. (2018). A water-soluble polysaccharide from the roots of *Polygala tenuifolia* suppresses ovarian tumor growth and angiogenesis in vivo. Int. J. Biol. Macromol..

[97] Ren F., Wu K., Yang Y., Yang Y., Wang Y., Li J. (2020). Dandelion polysaccharide exerts anti-angiogenesis effect on hepatocellular carcinoma by regulating VEGF/HIF-1α expression. Front. Pharmacol..

[98] Ke C., Li Z. (2021). Preparation, preliminary characterization and antiangiogenic activities of polysaccharides from pomegranate peels. J. Chem. Soc. Pak..

[99] Joseph M.M., Aravind S.R., George S.K., Varghese S., Sreelekha T.T. (2013). A galactomannan polysaccharide from *Punica granatum* imparts in vitro and in vivo anticancer activity. Carbohydr. Polym..

[100] Thotambailu A., Cheriamane D., Santhepete M., Kumar Bhandary S., Avanippully J., Bhadravathi P., Lagouri V. (2022). Role of pomegranate in the management of cancer. Pomegranate.

[101] Sartippour M.R., Seeram N.P., Rao J.Y., Moro A., Harris D.M., Henning S.M., Firouzi A., Rettig M.B., Aronson W.J., Pantuck A.J. (2008). Ellagitannin-rich pomegranate extract inhibits angiogenesis in prostate cancer in vitro and in vivo. Int. J. Oncol..

[102] Dana N., Javanmard S.H., Rafiee L. (2015). Antiangiogenic and antiproliferative effects of black pomegranate peel extract on melanoma cell line. Res. Pharm. Sci..

[103] Dana N., Javanmard S.H., Rafiee L. (2016). Role of peroxisome proliferator-activated receptor alpha and gamma in antiangiogenic effect of pomegranate peel extract. Iran. J. Basic Med. Sci..

[104] Consoli V., Burò I., Gulisano M., Castellano A., D’Amico A.G., D’Agata V., Vanella L., Sorrenti V. (2023). Evaluation of the antioxidant and antiangiogenic activity of a pomegranate extract in BPH-1 prostate epithelial cells. Int. J. Mol. Sci..

[105] Gul H.F., Ilhan N., Ilhan N., Ozercan I.H., Kuloglu T. (2021). The combined effect of pomegranate extract and tangeretin on the DMBA-induced breast cancer model. J. Nutr. Biochem..

[106] Mandal A., Bhatia D., Bishayee A. (2017). Anti-inflammatory mechanism involved in pomegranate-mediated prevention of breast cancer: The role of NF-κB and Nrf2 signaling pathways. Nutrients.

[107] Ahmadiankia N. (2019). Molecular targets of pomegranate (*Punica granatum*) in preventing cancer metastasis. Iran. J. Basic Med. Sci..

[108] Ahmadiankia N., Bagheri M., Fazli M. (2018). Gene Expression Changes in Pomegranate Peel Extract-Treated Triple-Negative Breast Cancer Cells. Rep. Biochem. Mol. Biol..

[109] Banerjee N., Kim H., Talcott S., Mertens-Talcott S. (2013). Pomegranate polyphenolics suppressed azoxymethane-induced colorectal aberrant crypt foci and inflammation: Possible role of miR126/VCAM-1 and miR-126/PI3K/AKT/mTOR. Carcinogenesis.

[110] Wang L., Alcon A., Yuan H., Ho J., Li Q.J., Martins-Green M. (2011). Cellular and molecular mechanisms of pomegranate juice-induced anti-metastatic effect on prostate cancer cells. Integr. Biol..

[111] Toi M., Bando H., Ramachandran C., Melnick S.J., Imai A., Fife R.S., Carr R.E., Oikawa T., Lansky E.P. (2003). Preliminary studies on the anti-angiogenic potential of pomegranate fractions in vitro and in vivo. Angiogenesis.

[112] Moga M.A., Dimienescu O.G., Bălan A., Dima L., Toma S.I., Bîgiu N.F., Blidaru A. (2021). Pharmacological and Therapeutic Properties of *Punica granatum* Phytochemicals: Possible Roles in Breast Cancer. Molecules.

[113] Bose A.S.C. (2017). Anti-Angiogenic and Vasculoprotective Effect of Punica Granatum Root. Master’s Thesis.

